# Radical Cure of Hydrocele, without Injections

**Published:** 1855-03

**Authors:** A. S. Hudson


### RADICAL CURE OF HYDROCELE WITHOUT INJECTIONS.

BY A. S. HUDSON, M. D.

Nearly a year ago, my attention was called to a case of hydrocele of the left side of the scrotum. For three months only palliative treatment was adopted, and by puncture the sac was four times emptied of its serous contents.

The patient, having listened to a lively picture of suffering which a neighbor had sustained while under treatment for this disease at Chicago, became prejudiced against the mode of treatment with irritating injections. He looked upon it with terpidation; and was, therefore, solicitous for some final though less formidable therapeutic plan.

Accordingly he was furnished with the ointment of tart. ant., and directed to courageously apply it to the diseased part until prominent pustules appeared. The first crop covered less than half of the tumid surface. These were allowed to run their course and decline. Then other remaining portions of the surface were topically medicated, and at length eroded. The lowest part of the scrotum resisting, the ointment was here lastly applied.

Altogether ten days or two weeks were consumed in this cautious tentative medication; yet it worked well. The artificial inflammation dipped deep, and caused the two surfaces of the tunic to adhere firmly together; and now for more than six months the patient has enjoyed a *useful* gland and a sound scrotum.

It may be remarked, that two weeks prior to the adoption of the above-mentioned measures, he was treated for inflamed testicle, which, from exposure to atmospheric vicissitudes, followed the last tapping. The reduced inflammation left an enlarged testicle surrounded with a little water. With this state of the parts the inunction was commenced. The tumid gland lessened with the fluid's disappearance.—*American Jour. of Med. Sciences.*

